# Purcell effect and Lamb shift as interference phenomena

**DOI:** 10.1038/srep20599

**Published:** 2016-02-10

**Authors:** Mikhail V. Rybin, Sergei F. Mingaleev, Mikhail F. Limonov, Yuri S. Kivshar

**Affiliations:** 1 Ioffe Institute, St. Petersburg 194021, Russia; 2 Department of Nanophotonics and Metamaterials, ITMO University, St. Petersburg 197101, Russia; 3 VPI Development Center, Belarus Hi-Tech Park, Minsk 220037, Belarus; 4 Nonlinear Physics Center, Australian National University, Canberra ACT 0200, Australia

## Abstract

The Purcell effect and Lamb shift are two well-known physical phenomena which are usually discussed in the context of quantum electrodynamics, with the zero-point vibrations as a driving force of those effects in the quantum approach. Here we discuss the classical counterparts of these quantum effects in photonics, and explain their physics trough interference wave phenomena. As an example, we consider a waveguide in a planar photonic crystal with a side-coupled defect, and demonstrate a perfect agreement between the results obtained on the basis of quantum and classic approaches and reveal their link to the Fano resonance. We find that in such a waveguide-cavity geometry the Purcell effect can modify the lifetime by at least 25 times, and the Lamb shift can exceed 3 half-widths of the cavity spectral line.

The Purcell effect^1^ and Lamb shift^2^ are among the frequently mentioned phenomena in quantum electrodynamics (QED). They both originate from the physics of the zero-point vibrations modifying the rate of spontaneous emission of a quantum particle and its transition energy. Although these effects have a great potential for many applications and being observed in a variety of different physical systems^3^,^4^,^5^,^6^,^7^,^8^,^9^,^10^,^11^,^12^,^13^, the fabrication of devices with proper quantum properties is a challenging task. On the other hand, recently an impressive progress was achieved in the development of technology and theory of classic structures such as photonic crystals and metamaterials composed of artificial meta-atoms with unique properties, including strongly modified local density of states (LDOS), negative permittivity 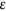 and permeability 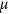, and engineered values of the refractive index 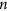
14. Such a progress in electrodynamics of complex photonic media suggests that many ideas and concepts usually discussed for quantum mechanics appear to be important in the study of wave physics being applicable to waves of different nature. The quantum-optical analogies involve scattering of particles with fixed angular momentum^15^ and Mie scattering^16^, Fano resonances in atomic physics^17^ and nanophotonic structures^18^,^19^,^20^,^21^,^22^, the concept of band structure of photonic crystals^23^, and many other more specific effects^24^. Also we notice the classic description of emission efficiency of dipole radio antenna^25^ and the recent papers on nanoantennas^26^,^27^. However, in all previous papers devoted to the classical description of the Purcell effect in antennas the authors studied oscillations of a charged particle or a loop current. In contrast, here we consider a pure photonic mode in a microcavity to reveal a direct analogy of these QED effects with the classical wave theory for the modes which create a base of photonic on-chip devices.

Here we perform a detailed analysis of the Purcell effect, Lamb shift, as well as more familiar Fano resonance for two problems: (i) the problem of a quantum particle in a cavity, and (ii) a classical problem of the wave propagation of a photonic-crystal waveguide with a Fabry-Perot resonator (FPR) and a side-coupled defect. We demonstrate a close analogy between these different types of interference phenomena and reveal similarity of the resulting analytical formulas. Finally, we verify our results for the Purcell effect and Lamb shift of the classical system by means of direct numerical calculations of a photonic crystal structure based on classic Maxwell’s equations. We demonstrate that in the waveguide-cavity structure the Purcell effect can modify the radiation lifetime at least by 25 times, and the Lamb shift can exceed 3 half-widths of the cavity spectral line.

First, we remind general results for a system consisting of a quantum particle (say an atom) placed in the middle of a resonator [see Fig. 1(a)]. For such a geometry, the QED approach predicts two effects^28^. The first one is the Purcell effect related to the fact that the photonic LDOS differs from the vacuum density of states, that results in the enhancement (or suppression) of the rate of spontaneous emission. The second effect is the Lamb shift, being simply a shift of the transition energy due to the perturbation of stationary modes by the zero-vibrations of electromagnetic field.

For more specific example, here we study a waveguide-cavity structure with a side-coupled defect shown schematically in Fig. 1(b). A waveguide confines the propagating light in two directions and it has two partially reflective elements that play a role of two mirrors of an effective resonator. A two-level atom is introduced near the waveguide for the quantum case or a cavity with a narrow band as a meta-atom for the classic case. Such a structure was studied for different designs including photonic crystals^29^,^30^, micro-ring resonators^31^,^32^, whispering-gallery modes^33^,^34^, as well as for structures with quantum dots^35^,^36^. In addition, the vacuum Rabi splitting of atoms in similar systems was described in a classical model^37^.

## Results

### Quantum approach

First, we discuss the QED approach based on the Jaynes-Cummings model with the Wigner-Weisskopf approximation^28^,^38^. The Hamiltonian of a two-level atom (or a quantum dot) interacting with the electromagnetic field can be presented in the form,


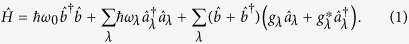


where 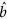, 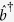 and 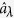, 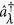 are the annihilation and creation operators for atomic and electromagnetic states, respectively, 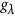 describes a coupling between an electron and an optical mode 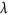. We notice that this description has a number of limitations, e.g. (i) the analysis of a two-level system with bosonic operators is only valid when saturation effects are neglected; (ii) the lifetime of the optical FPR mode is much less than that of a two-level system without FPR.

By solving Heisenberg’s equation, we calculate the spontaneous decay rate^39^


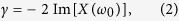


and the Lamb shift,


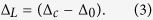


Here 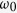 is atomic frequency in free space, 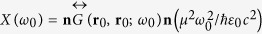, 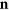 is an unit vector directed toward an atomic dipole moment 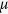, 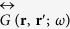 is the dyadic Green’s function, 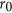 is a position of the atom. The vacuum part of the Lamb shift, 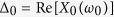, where 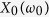, is related to the atom in vacuum (the two-level system should be specified to evaluate 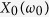), and the cavity-associated part is 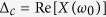.

**Figure 1:**
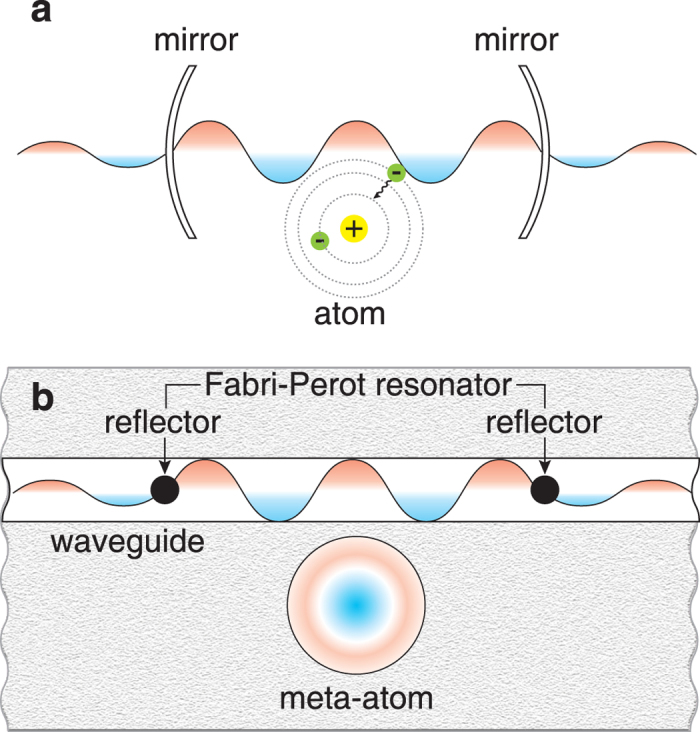
Schematic of quantum and classical systems demonstrating the Purcell effect and Lamb shift. In the case (**a**) a quantum particle is placed into a resonator. In the case (**b**) a photonic cavity is placed in a close proximity to a FPR waveguide in a planar photonic crystal being described by Maxwell’s equations.

The system under study is shown in the Fig. 1b. We assume that the waveguide supports only one mode in each direction. Therefore, the FPR forms effectively one-dimensional cavity. We use Green’s function of the system with partially reflective elements characterized by the reflection coefficient 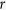 (see Methods for details)


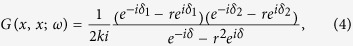


where 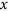 is a point between the reflectors, 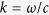 is the wavenumber, 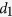 and 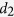 are the distances between the point 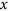 and left/right reflector, respectively, 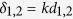, and 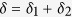.

If we define the vacuum rate of spontaneous emission as 
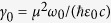
, the Purcell factor can be presented in the form,


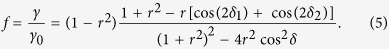


Similarly, the Lamb shift is found as


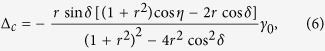


where 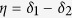.

**Figure 2:**
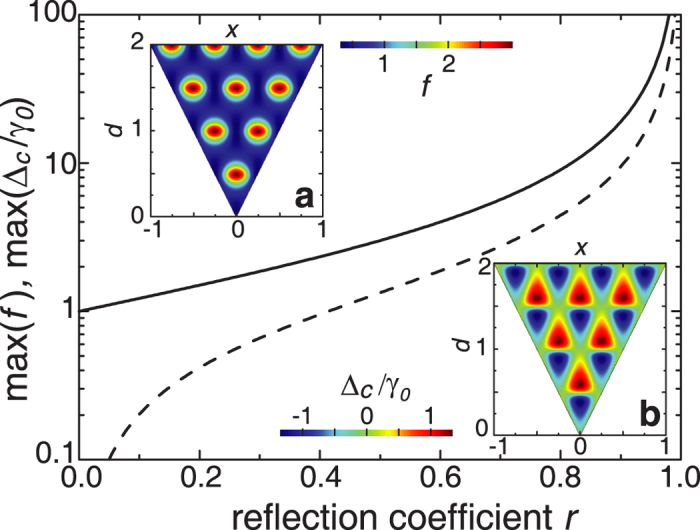
Purcell factor and Lamb shift. Maxima of the Purcell factor (solid curve) and Lamb shift (dashed curve) as functions of the reflection coefficient 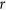 for the quantum system presented in Fig. 1a. Inserts: (**a**) Purcell factor for different positions 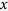 of an atom vs. 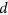 in the units of 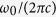. (**b**) Lamb shift for different positions 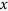 vs. 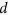. Both inserts are shown for 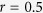. The point 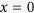 corresponds to the center of the FPR.

Figure 2 shows the maxima of the Purcell factor and Lamb shift for a small quantum particle located inside FPR. The maximum of the Purcell factor is calculated as


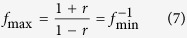


and the maximum of the Lamb shift


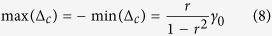


is proportional to the linewidth of an atom radiation in vacuum being small for a quantum system with a large enough lifetime. In contrast, the Purcell effect changes dramatically the rate of spontaneous emission; for example, when 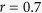 this change, defined as the ratio 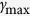 to 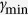, is more than 30 times. Inserts in Fig. 2 demonstrate the Purcell factor and Lamb shift as functions of two variable: the particle position 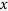 in FPR and the distance 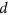 between the reflectors.

We may transform the expression for the Purcell factor to a traditional form^1^. Indeed, when we consider the case 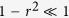 for the system where the resonance frequency of an atom is tuned to the FPR mode and the atom is placed in the field antinode to maximize the rate of spontaneous emission. The Purcell factor can be written in the form


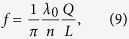


where 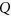 is the quality factor (see Methods), 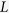 is the FPR length, 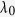 is the vacuum wavelength, and 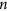 is an effective mode index of the waveguide.

### Classical approach

For the classic counterpart of a quantum system we consider a microcavity, which we refer to as a meta-atom instead of a quantum particle. We introduce two subsystems, namely a low-Q FPR associated with a pair of partially reflecting defects and a meta-atom characterized by a narrow Lorentzian spectrum. Although such classic systems are discussed in many papers^31^,^32^,^33^,^34^,^35^,^36^ in relation to the Fano resonance, to the best of our knowledge, both the Purcell effect and Lamb shift were not discussed for such geometries.

For the structure presented in Fig. 1(b), we find the transmission spectrum by means of the transfer matrix approach. For the case 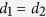, we obtain^29^


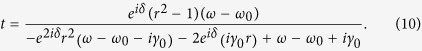


We then extract the Lorentz function from Eq. (10)


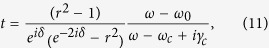


where


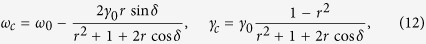


and finally we present the transmission intensity through the Fano formula


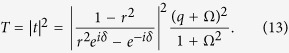


Here 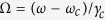 is the dimensionless frequency, and 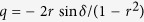 is the Fano parameter. Now we can draw an important conclusion that eq. (12) contains exactly the same expressions for the Purcell factor as the QED formulas (5) while the expression for the Lamb shift differ by the factor 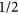 (this difference has been discussed in literature^40^). Besides, we find a linear relation between the Purcell factor and Lamb shift on the Fano parameter


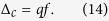


We notice that eq. (13) shows that the transmission intensity is determined by two terms. The first term governs the transmission in the absence of the meta-atom (FPR only), and the second term describes the interaction with the meta-atom through the Fano interference.

### Numerical results

To illustrate the results of our analytical study, we consider the propagation of 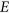-polarized (i.e. described by the electrical field 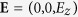) light in a popular photonic crystal structure^29^. Specifically, we assume that the photonic crystal is made up of a square lattice of cylindrical dielectric rods with a refractive index of 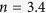 (which corresponds to silicon in IR) and a radius of 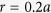, where 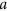 is the lattice spacing. A waveguide is formed by removing a row of dielectric rods, and a cavity (a “meta-atom”) is created by reducing the radius of a single rod to 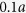. The cavity is placed at the distance 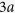 away from the waveguide. Such a cavity supports a localized monopole-like defect mode with the resonant frequency 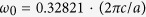 and the half-width at half minimum 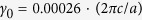. To form the required FPR structure, two identical partial reflectors (cylinders of radius 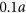 made of the same material as all other rods) are placed in the waveguide symmetrically around the meta-atom. The transmission spectra in this structure are calculated by employing the frequency-domain Wannier functions approach^41^, which allows to match the waveguide modes more efficiently and exclude parasitic back reflections from photonic crystal boundaries of the simulated structure than in the finite-difference time-domain (FDTD) method^29^, and thus enables accurate modeling of high-Q resonances. The use of 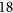 maximally localized Wannier functions ensures accurate results for any (including fractional with respect to 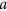) distances 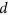 between the FPR reflectors.

**Figure 3:**
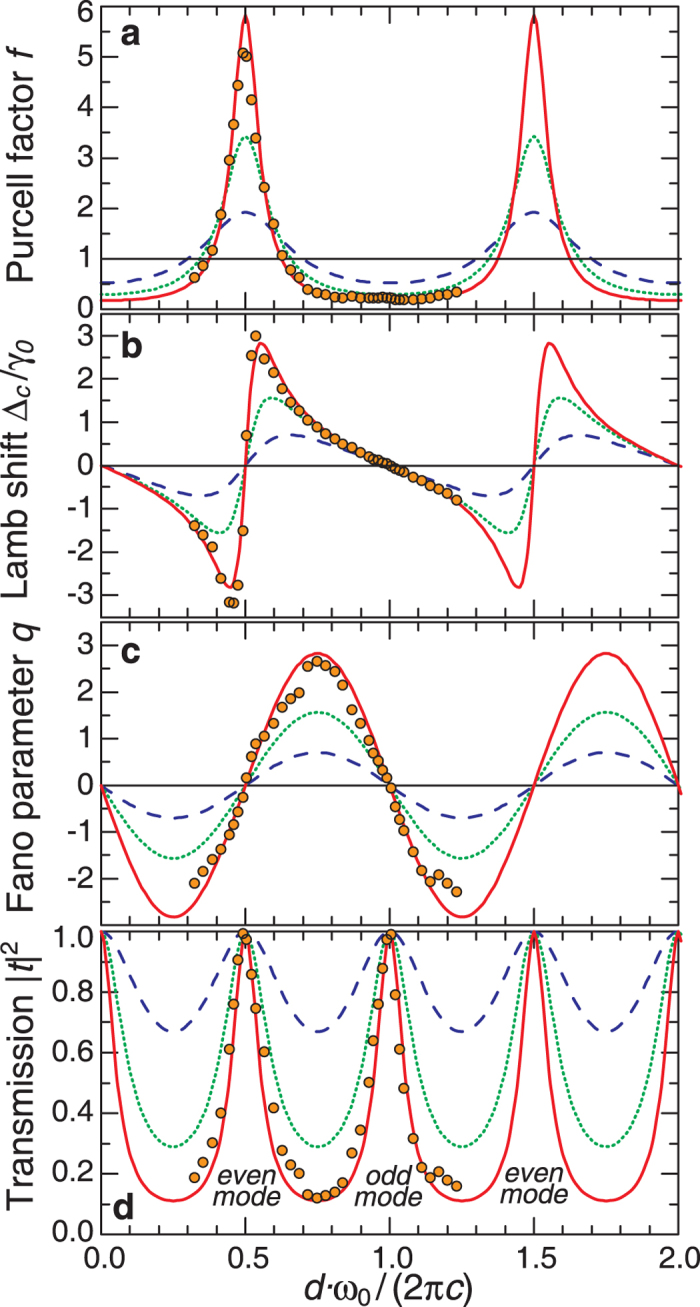
Results of our analysis of the photonic structure with a side-coupled defect. (**a)** Purcell factor, (**b**) Lamb shift, (**c**) Fano parameter, and (**d**) transmission coefficient. Curves are calculated for 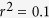 (dashed blue), 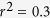 (dotted green) and 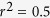 (solid red). Circles are the values obtained from fitting of the spectra calculated directly for the photonic crystal circuit.

We calculate the dependence of the transmission spectra in our structure vs. the distance between FPR reflectors by varying the distance from 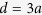 to 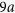 with a step of 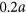. Each spectrum has been fitted by eq. (13) taking into account that due to inhomogeneity of photonic crystal waveguide 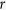 becomes a function of 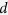. To simplify such a fitting, we also calculate the transmission spectra for the same FPR structures but without meta-atom, which correspond to the first multiplier on the right hand side of eq. (13). This allows to extract the second multiplier in eq. (13) and evaluate 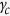, 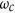 and 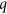. Figure 3 shows the corresponding extracted Purcell factor, Lamb shift, and Fano parameter in comparison with the dependences predicted by the analytical theory. As can be seen, the results of ab-initio calculations are in an excellent agreement with the analytical data (small deviations are explained by the dependence of 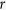 on 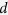, which is ignored in the simplified analytical model).

Figure 3 shows two extreme cases related to the FPR modes with different parity. Odd FPR modes correspond to the small Lamb shift and Purcell factor less then unity (low decay rate) while even FPR modes correspond to a strong Lamb shift and larger values of Purcell factor (short lifetime).

## Discussion

**Figure 4:**
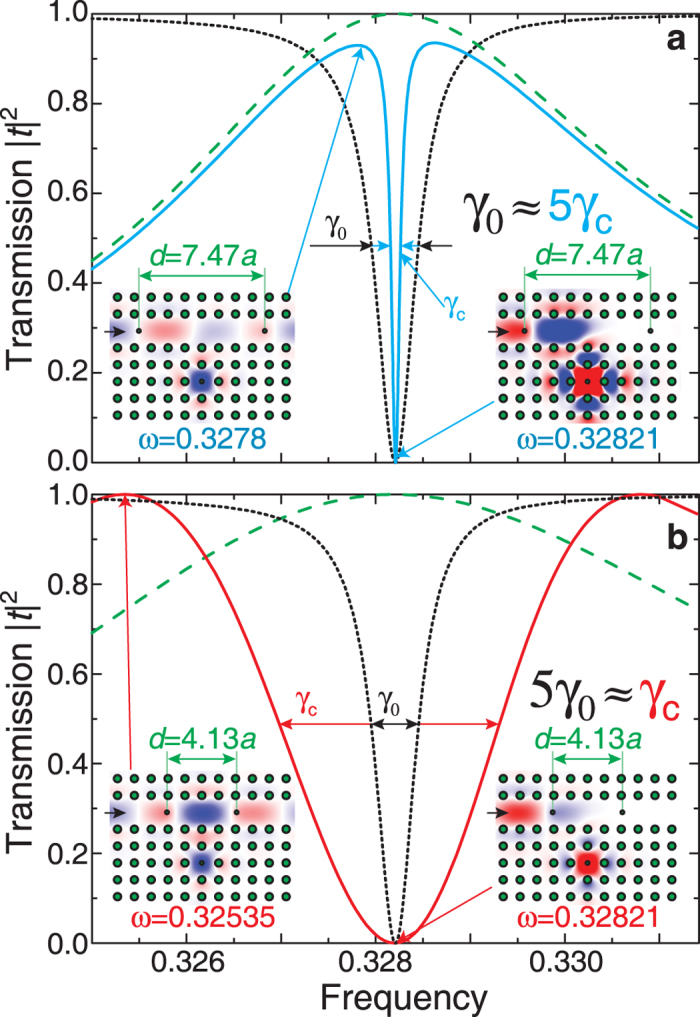
Manifestation of the Purcell effect in photonic-crystal waveguide with a cavity. (**a**) An increase of the lifetime. The transmission spectrum of the structure without FPR (black dotted curve) 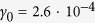 and FPR only (green dashed curve) at 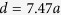. The spectrum of the complete structure is shown by a blue solid curve, 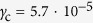. (**b**) A decrease of the lifetime. The transmission spectrum of the structure without FPR (black dotted curve) 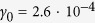 and FPR only (green dashed curve) at 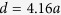. The spectrum of the complete structure is shown by a red curve, 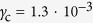. Inserts show the 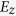 field distributions for the indicated frequency values.

We now compare the results for the Purcell effect and Lamb shift calculated in terms of the QED approach and the classical electrodynamics. For the case of odd modes, within the QED approach we evaluate LDOS being rather small. We argue that the overlap integral vanishes because the parity of the meta-atom mode is even. In the classical approach, our description is based on the energy conservation principle. The meta-atom emits light which is reflected and come back to an emitter. This reflected radiation additionally excites the meta-atom, and this process returns a part of the emitted energy back to the meta-atom. As a result, the decay rate is decreased, and the line width of radiation becomes narrow. In a photonic-crystal circuit with FPR shown in Fig. 4(a), we obtain an increase of the 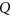-factor in 4.5 times. We notice that the maximum lifetime is increasing, and we observe a growth in 5.42 times for 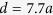. For this case, the Fano parameter is 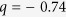, and the line shape is asymmetric.

For even FPR modes, the Purcell factor has high values because of large LDOS, and larger overlap integral since both modes are even. However, the classical approach applied to this effect seems counterintuitive. Indeed, the issue is how the FPR draws out the field from the meta-atom. We notice that QED predicts that the Lamb shift is observed when an atom and environment are coupled [see Fig. 3(b)].

To describe this effect, we should take into account the superposition principle that manifests itself as an interference phenomenon. We assume that the meta-atom is excited in an initial mode. When it emits light, a portion of radiation returns back from the reflector. To proceed further we should take into account the phase of the reflected wave. Indeed, the reflected light can excite the meta-atom mode out of phase and due to the superposition principle the initial mode and the mode excited by a reflected wave have to interfere. The destructive interference leads to a decrease of the resulting mode amplitude with the corresponding energy stored in the meta-atom. Therefore, this process gives rise to an enhancement of the radiation decay rate and broadening of the line width. As a result, the lifetime is decreased by the factor 5, as shown in Fig. 4(b).

Returning back to the discussion of the Lamb shift, we notice that the mode excited by a reflected wave is out of phase so that it can modify essentially the resulting amplitude such that being retarded or advanced in the time domain respective to non-perturbed initial mode and the oscillation period should increase or decrease, respectively. Hence, interference with out-of-phase reflected wave will lead to a frequency shift.

A control of the decay rate has a strong potential for applications. Here we reveal that the lifetime is varied by 25 times with changing from even to odd mode of FPR. This property can be useful, for example, for realizing an optical memory device. First, we prepare a meta-atom in a low-Q mode. The meta-atom receives the light pulse. Next, when the FPR parameters are changed externally in such a way that the FPR mode becomes odd. As a result, light is trapped in the meta-atom. To modulate the properties of the FPR it is not necessary to change the geometry of the device. Instead of this the reflectors can be made from a material, which properties are modified by ultrafast all-optical switching. We notice the lifetime of the meta-atom is varied by the working frequency as well as Q-factor of the meta-atom without FPR environment.

We have shown that the Purcell effect and Lamb shift can be introduced in the framework of classic electrodynamics by employing the concept of an effective “meta-atom” interacting with an optical resonator. In the quantum case, an atom releases its energy due to interaction with the zero-point vibrations, and the vacuum photonic LDOS is evaluated by means of Green’s function obtained from Maxwell’s equations. In the classical case, a meta-atom releases energy without any vacuum fluctuations, however the radiated field excites the modes of a low-Q resonator being described with the same Green’s function as in the quantum case. As a result, for both classic and quantum systems the effects become similar.

By employing our approach, we can get a deeper physical insight into such phenomena as the Purcell enhancement, Lamb shift, and Fano resonance that can be identified in the calculated or measured spectra of a classical radiating photonic system. When a narrow-band mode (a system characterized by a discrete eigenmode) is superimposed with a broadband background radiation (a continuum spectrum of eigenmodes), we can expect the existence of *three distinct interference effects*: (i) a change of the radiation rate caused by the Purcell effect; (ii) a change of the resonance frequency associated with the Lamb shift, and (iii) an asymmetric line-shape explained by the physics of the Fano resonances.

We believe these results may open a new way for realizing classical analogues of the Purcell effect and Lamb shift in much simpler photonic structures than the systems involving quantum emitters. Moreover, the physics of photonic crystals and metamaterials do not require low temperatures and high-precision measurements usually attributed to quantum systems. We expect that other remarkable quantum phenomena may find their analogues in the physics of classical nanophotonic structures.

## Methods

### Analysis of the field in the waveguide

Here we consider a waveguide that supports only one mode in each direction being described by the homogeneous one-dimensional Helmholtz equation


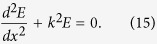


The equation (15) has two linearly independent solutions 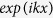 and 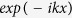. Hence the electromagnetic field can be described by a pair of amplitudes 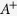 and 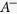 as 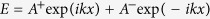. If the field is known at point 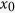, we can calculate the field at any other point 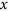 by means of a propagating transfer matrix


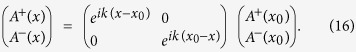


To describe inhomogeneities at the point *x*′, we connect the field amplitudes at one edge with the other by the transfer matrix


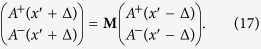


Here we assume that the length of inhomogeneity is 2Δ. For a reflector, it is convenient to choose the edges in such a way that the transfer matrix has a form^29^


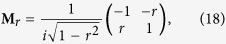


where the reflection coefficient 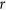 is a real parameter in the interval 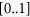.

To calculate the transmission spectra trough the waveguide with FPR, we multiply three matrices and find the transmission coefficient with its approximation at the 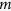-th resonance (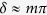)


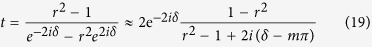


and evaluate the quality factor


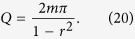


### The Green function

Now we can find Green function for the waveguide with FPR. The Green function for the homogeneous eq. (15) read^42^


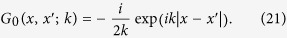


We notice this Green function satisfies the equation for the Green function if 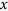 in the homogeneous region. However 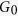 does not obey boundary conditions, namely Green function has not to contain the incoming waves traveling toward the FPR. We construct the Green function by adding to 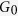 a solution of the 1D Helmholtz equation. Below we discuss the case of the 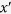 is restricted by the FPR. Therefore the Green function can be described by an amplitudes 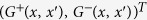. We connect two points 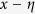 and 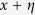 by the matrix relation


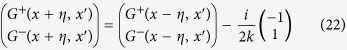


in supposing that positive 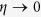. At other points 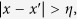 we can use the conventional transfer matrices (16) and (18) to find 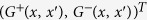. By setting 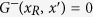 at the right boundary of the FPR and 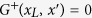 at the left we can obtain 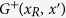 and 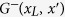 amplitudes. Using the transfer matrices we yield desired Green’s function as a sum of the amplitudes 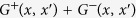 at any point 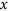, including 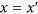 that was written in Eq. (4). We also notice that they are the matrix elements of the dyadic Green function, which has a diagonal form in the case under study.

## Additional Information

**How to cite this article**: Rybin, M. V. *et al*. Purcell effect and Lamb shift as interference phenomena. *Sci. Rep.* **6**, 20599; doi: 10.1038/srep20599 (2016).
